# Epidermolysis Bullosa-Associated Squamous Cell Carcinoma: From Pathogenesis to Therapeutic Perspectives

**DOI:** 10.3390/ijms20225707

**Published:** 2019-11-14

**Authors:** Angelo Giuseppe Condorelli, Elena Dellambra, Elena Logli, Giovanna Zambruno, Daniele Castiglia

**Affiliations:** 1 Genetics and Rare Diseases Research Division, Bambino Gesù Children’s Hospital, IRCCS, viale di San Paolo 15, 00146 Rome, Italy; 2 Laboratory of Molecular and Cell Biology, IDI-IRCCS, via Monti di Creta 104, 00167 Rome, Italy

**Keywords:** cancer, wound-healing, basement membrane zone, extracellular matrix, fibrosis, inflammation, immunity, collagen VII, laminin-332, kindlin-1

## Abstract

Epidermolysis bullosa (EB) is a heterogeneous group of inherited skin disorders determined by mutations in genes encoding for structural components of the cutaneous basement membrane zone. Disease hallmarks are skin fragility and unremitting blistering. The most disabling EB (sub)types show defective wound healing, fibrosis and inflammation at lesional skin. These features expose patients to serious disease complications, including the development of cutaneous squamous cell carcinomas (SCCs). Almost all subjects affected with the severe recessive dystrophic EB (RDEB) subtype suffer from early and extremely aggressive SCCs (RDEB-SCC), which represent the first cause of death in these patients. The genetic determinants of RDEB-SCC do not exhaustively explain its unique behavior as compared to low-risk, ultraviolet-induced SCCs in the general population. On the other hand, a growing body of evidence points to the key role of tumor microenvironment in initiation, progression and spreading of RDEB-SCC, as well as of other, less-investigated, EB-related SCCs (EB-SCCs). Here, we discuss the recent advances in understanding the complex series of molecular events (i.e., fibrotic, inflammatory, and immune processes) contributing to SCC development in EB patients, cross-compare tumor features in the different EB subtypes and report the most promising therapeutic approaches to counteract or delay EB-SCCs.

## 1. Introduction

**Figure 1:**
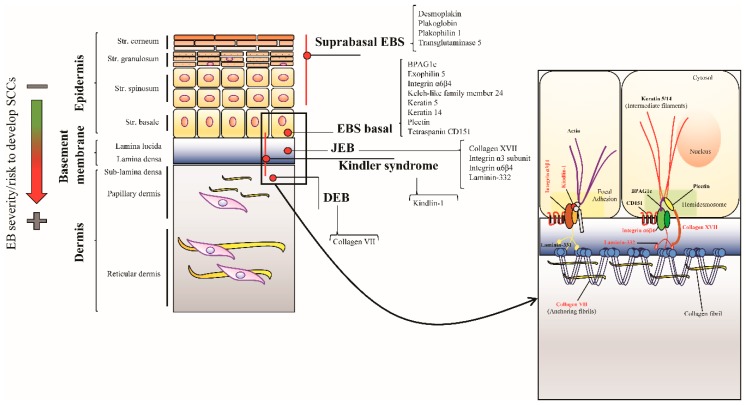
Schematic representation of the epidermis depicting levels of cleavage sites and mutated proteins for each epidermolysis bullosa (EB) type. Epidermal cells layers, from the stratum basale to the stratum corneum (flat, orange boxes), and the underlying papillary and reticular dermis are depicted. Basal keratinocytes are attached to the dermis by multiprotein complexes linking keratin intermediate filaments to anchoring fibrils through hemidesmosomes and the epithelial laminin isoform, laminin-332. Focal adhesions also contribute to stabilizing the cutaneous basement membrane zone (BMZ). The skin level where blisters arise in each epidermolysis bullosa (EB) type (red lines and dots), and the corresponding mutated proteins are indicated. Inset magnification shows the BMZ, with proteins mutated in Kindler syndrome, junctional EB (JEB) and dystrophic EB (DEB) shown in red. In EB forms compatible with survival to adulthood, the risk of cutaneous squamous cell carcinoma (SCC) occurrence correlates with EB severity (green arrow turning into red. Green = low/mild severity EB type and a low risk to develop SCC, red = severe EB type and high risk to develop SCC).

Inherited epidermolysis bullosa (EB) comprises a clinically and genetically heterogeneous group of rare genetic diseases characterized by skin fragility and blister formation following minor trauma [1]. Four major EB types are distinguished based on the level of blister formation within the skin: EB simplex (EBS), junctional EB (JEB), dystrophic EB (DEB), and Kindler syndrome (KS) (Figure 1). According to the current “onion skin” classification approach, each EB type then comprises several subtypes characterized by different modes of inheritance, causative genes and mutations, phenotypic and molecular features [1]. EB clinical spectrum ranges from early-lethal forms with extensive cutaneous and extracutaneous involvement to mild phenotypes with localized skin lesions only. Disease-causing variants in at least 20 different genes account for the genetic heterogeneity of EB [2] (Figure 1).

In recessive DEB, JEB and KS blistering occurs within or below the cutaneous basement membrane zone (BMZ) and leads to chronic wounds with fibrosis and inflammation healing with scarring sequelae. These patients have an increased risk to develop one or more cutaneous squamous cell carcinomas (SCCs) [1]. Usually, the tumors are localized at sites of chronic, hard-to-heal wounds and scarring, they are frequently multiple, and, specific to recessive DEB, highly aggressive representing the first cause of death in this EB subtype. Though the age of onset, cancer localization and carcinogenetic processes may be to some extent different across the various EB (sub)types, EB-related SCC (EB-SCC) represents an important model towards a more complete understanding of mechanisms responsible for carcinogenesis occurring within a fibrotic and inflamed microenvironment. In this review, we will discuss SCC clinical and molecular features in each cancer-prone EB type, focusing on the latest findings unveiling novel and potentially relevant pathomechanisms underlying tumor development in EB patients and highlighting gaps in the current literature. In addition, the most promising direct and indirect therapeutic strategies to counteract EB-SCC, with special reference to the devastating SCCs occurring in recessive DEB, will be addressed.

## 2. SCC in the General Population

Cutaneous SCC (hereafter indicated as SCC) represents the second most common non-melanoma skin cancer (NMSC) after basal cell carcinoma (BCC). While the incidence ratio of BCC to SCC is traditionally considered to be 4:1, a recent study reported an age-weighted incidence ratio of 1.4:1 in the USA population [3]. However, statistics on the incidence of SCC are difficult to calculate because of limited data, due to incomplete registration practices in many countries or, as in the USA, to the absence of a national SCC registry. According to a recent study, the age-standardized incidence rate of the first SCC in the UK population in the period 2013–2015 is equal to 77 cases per 100,000 person-years (PY) [4]. All reports agree that the risk to develop SCC (i) increases progressively with patient age and reaches its maximum after 60 years, (ii) is highly affected by the amount of ultraviolet (UV) irradiation, in relation to country latitude and skin phototype of inhabitants, and (iii) is growing with time, probably due to the worldwide population ageing and improved screening procedures [4,5]. Similar to incidence data, mortality rates are not well documented, but are estimated to be around 0.41–0.52 per 100,000 PY for UV-induced SCC (UV-SCC), and 4.94 per 100,000 PY for SCC in organ transplant recipients in USA population [6].

The major risk factors for SCC onset are chronic exposure to UV radiation, immunosuppression in solid organ transplants recipients and human papilloma virus (HPV) infections [5]. However, chronic exposure to UV rays is certainly the main driver of SCC as shown at an epidemiologic and molecular level. Gene mutations are strongly enriched for C > T transitions at dipyrimidine-sites, the peculiar mutation signature of UVB radiation. In patients with SCC, mutations in the tumor suppressor gene *TP53* are considered the most frequent, but genetic alterations in other cancer-related genes, such as cyclin-dependent kinase inhibitor 2A (*CDKN2A*), *NOTCH1/2* and *RAS*, as well as chromosomal rearrangements have been found [7]. UV-SCC arises from a multistep process of accumulation of genetic hits, due to cumulative UV exposure.

Actinic keratosis (AK) is the most common in situ cancerous skin lesion that has not yet acquired the full complement of mutations and invasive growth characteristics associated with SCC [8]. Although the presence of AKs in photodamaged areas is considered one of the best predictors of SCC development, only a limited number of AKs progresses to SCC [9]. The genetic profile of AKs is not completely predictive of their evolution as mutations in typical tumor suppressor genes, mainly *TP53*, and other genetic alterations are shared with cutaneous SCCs [10,11]. Most cutaneous SCCs have a good prognosis if diagnosed and treated early by complete surgical excision. Only a subset of SCCs behaves aggressively with a high risk of recurrence and metastasis; these unusual tumor features are due to localization in high risk body areas, depth (>2 mm thickness), size, and histologic features (poor differentiation), as well as patient characteristics and comorbidities [5,12].

## 3. Wound Healing and SCC

In the general population, alongside the most common risk factors underlying SCC development, severely burned areas and hard-to-heal, chronic wounds (e.g., long-standing venous stasis ulcers) represent susceptibility sites for the development of a rare and aggressive form of skin cancer, defined in the early 1800s as Marjolin ulcer (MU). Although MU embraces a histologically heterogeneous set of malignancies, well-differentiated SCCs account for the majority of them (≈ 70% of cases). Of note, MU-SCCs are more aggressive than skin SCCs of different etiology, and metastasize in more than 27% of cases [13]. Though a number of theories have been proposed to explain the relation between skin damage and MU development [13], a common denominator is the complex series of events determined by the derailed/prolonged wound healing process at the lesional area [14,15]. Indeed, the aberrant activation of wound healing pathways is a relevant, well-known event able to establish an inflamed, fibrotic stroma which represents the scaffold for tumor initiation and progression [14,16]. In this regard, the connection between the risk of occurrence of epithelial cancer and the unremitting and altered wound healing process of severe EB patients is clear-cut. Despite the primary genetic defects underlying each inherited EB type, all EB patients share skin and/or mucosal fragility and blistering. However, in the most severe and disabling EB (sub)types mutations in genes coding for specific components of the epidermal-dermal junction strongly compromise the healing process, and, in turn, skin erosions and blisters evolve in chronic wounds, inflammation and fibrosis [17]. These events are responsible for the onset of highly disabling and even life-threatening disease complications, such as esophageal strictures, deformities of hands and feet, and aggressive epithelial cancers [18]. 

## 4. Dystrophic EB

### 4.1. Clinical Features

DEB is the second most common EB type, and the most disabling one. The estimated prevalence of DEB ranges from approximately 6 per million in the USA and Spain to 20 per million in Scotland, the latter probably reflecting greater capture rather than a true higher prevalence [19,20,21]. DEB is caused by mutations in the *COL7A1* gene that encodes collagen VII (COL7), the major component of anchoring fibrils, ensuring adhesion of stratified epithelia to the underlying mesenchyme. Loss of the structural function of COL7 causes lifelong blistering and impaired wound-healing, leading to chronic wounds characterized by increased bacterial colonization, fibrosis and inflammation and to progressive scarring, which in turn can evolve as a systemic disease with secondary multiorgan involvement and propensity to early skin cancer development [1,17,22,23,24].

In particular, the recessive DEB subtype termed severe generalized (RDEB-SG) strongly predisposes patients to the development of multiple SCCs. RDEB-associated SCCs (RDEB-SCCs) are more aggressive than UV-SCCs in the general population and characterized by high morbidity and mortality: SCC represents the first cause of death in patients suffering from RDEB-SG. The cumulative risk of developing at least one SCC for patients with RDEB-SG increases with age, being already 67.8% by age 35 and attaining 90.1% by 55 years in the USA National EB Registry [25]. The risk of developing SCC is also increased in DDEB and in other RDEB subtypes, but they are less common than in severe RDEB and occur later in adulthood.

Typically, SCCs develop at sites of chronic wounds and scarring, in particular, the extremities [18,25]. Though the large majority of EB-SCC are histologically well-differentiated, they have a high propensity to local relapse and metastasis [18]. Early detection is relevant towards effective surgical excision, which remains the treatment of choice [26]. However, early diagnosis of SCC in RDEB patients remains a challenge, since the presence of numerous large chronic wounds and scar sites, together with a not straightforward choice of biopsy site, can require histopathologic evaluation of multiple biopsies [26]. In addition, by histopathology RDEB-SCC may be difficult to differentiate from granulation tissue or pseudoepitheliomatous hyperplasia [26]. All these criticalities contribute to the delay in diagnosis and management of RDEB-SCC. Late diagnosis and SCC aggressive features are the major determinants of the poor prognosis in these patients. Indeed, the cumulative risk of death from SCC in RDEB-SG who developed at least one SCC was 57.2% by age 35 and raised to 87.3% by age 45 in the USA National EB registry [25].

### 4.2. DEB-SCC Genetics

The skin is the body’s outermost barrier and represents the main target for a variety of external challenges, ranging from chemical to physical, mechanical and biological insults. As a result, genetic and epigenetic hits accumulate into the keratinocyte DNA as part of a physiological, naturally occurring process. In particular, the exposure to UV rays determines a specific signature of C > T and CC > TT mutations, which represent the majority of the somatic mutations in the skin [27]. However, UV-derived mutations do not necessarily lead to malignant transformation of keratinocytes in chronically sun-exposed skin areas [28].

This evidence highlights that the acquisition of the hallmarks of cancer [29] is a complex process where multiple mutation-dependent and independent events, such as the skin microenvironment, cooperate to determine tumor development and aggressiveness. In this respect, the case of RDEB-SCC molecular etiology is striking.

Although RDEB-SCC is typified by a surprisingly early age of onset and aggressiveness as compared to UV-SCC affecting non-RDEB patients, the genetic profiles of these tumors are quite similar and only partly explain their different features [30,31]. Indeed, whole-exome sequencing analyses revealed that RDEB-SCCs share with UV-SCCs a heterogeneous set of mutated genes and a number of cytogenetic alterations. In particular, RDEB-SCCs display mutations in *TP53, NOTCH1, NOTCH2, CDKN2A, HRAS*, and *FAT1*, a set of genes also mutated in aggressive cutaneous SCCs and considered as potential drivers of the tumor [32,33] (Table 1). The genetic landscape of RDEB-SCCs presents a high occurrence of inactivating mutations in NOTCH family members, suggesting a relevant role for the NOTCH pathway in RDEB keratinocyte transformation. Though loss-of-function mutations in *NOTCH1* are the most represented in RDEB patients and play a well-established role in mouse skin tumorigenesis [34], mechanistic studies in RDEB-SCC models are missing.

RDEB-SCCs show a very high mutational burden in relation to the early age of onset. Moreover, changes are not related to UV exposure. Recently, mutations in RDEB-SCC have been shown to be endogenously determined by the cytosine deaminase activity of APOBEC (apolipoprotein B mRNA editing enzyme, catalytic polypeptide-like) family of enzymes [31]. APOBEC enzymes are relevant gene editors in accordance to their ability to deaminate cytidines into uridines in 5′-TCW contexts (where W = A or T), which determinates C-to-T and C-to-G mutations (APOBEC signature). In RDEB-SCC, APOBEC activity is strongly enhanced and contributes to a high percent of mutations in typical RDEB-SCC-associated driver genes (e.g., *HRAS, NOTCH1, TP53*). Although the APOBEC signature has been found, to a different extent, in several cancer types, in RDEB-SCC the amount of mutations determined by APOBEC activity is significantly higher than that detected in non-RDEB SCCs (42% vs. 1.7–2%) and even in HPV-positive SCCs that have abundant APOBEC-driven mutations (≈ 30%) [7,31].

In physiological conditions, APOBEC-derived nucleotide changes are important in keratinocyte differentiation [35], lipid metabolism, adaptive immunity and anti-viral defense [36]. However, if dysregulated, APOBEC activity leads to genomic instability and contributes to cancer development. APOBEC members are over-expressed in RDEB-SCC and other cancer types [37] in response to several environmental factors, such as microbial insults and skin-injury dependent cell stress and inflammation. Interestingly, in RDEB patients, the up-regulation of APOBEC3A, APOBEC3B and APOBEC3H members is particularly prominent in areas of chronic tissue damage [31]. These findings expand our knowledge on RDEB-SCC pathomechanisms and could trigger the development of genomically-driven treatments, such as anti-APOBEC therapies.

### 4.3. DEB-SCC Microenvironment

#### 4.3.1. The Wound-Healing Process

The wound healing is a complex biological phenomenon able to re-establish tissue integrity after an injury. A plethora of cell-types, extracellular matrix (ECM) proteins, and soluble factors (cytokines, growth factors, hormones) are involved in a well-orchestrated cascade events that can be classically resumed in three sequential stages: (1) Inflammation, (2) new tissue formation, and (3) remodeling [38]. In the later “tissue formation” phase dermal fibroblasts and other precursors cells are stimulated to differentiate into a cell type called myofibroblast, typified by contractile and secretory abilities. Myofibroblasts are well-discriminated from tissue fibroblasts by production of specific contractile proteins (the “myo” attribute), such as α-smooth muscle actin (α-SMA) or transgelin (TAGLN), stress fibers assembly, and secretion of specific matricellular proteins, such as the fibronectin isoform ED-A, cellular communication network factor 2 (CCN2), periostin (POSTN) and tenascin-C (TNC) [39]. A central role in myofibroblast development and maintenance is played by the prototypic fibrotic cytokine, the transforming growth factor-β1 (TGF-β1), whose activation depends on ECM composition and mechanical state (matrix stiffness), as well as on ECM interaction with different cell-types, including the same myofibroblasts. Following wound re-epithelialization, myofibroblasts are physiologically cleared through cell death via apoptosis or de-activated and converted in a different cell lineage [40]. Finally, the complex processes underpinning wound healing must be finely tuned to be properly completed and to avoid pathological states as fibrosis.

#### 4.3.2. Fibrosis

In RDEB patients, fibrosis is a regular and severe disease complication resulting from the impaired healing of chronic wounds [41]. Dermal fibroblasts in RDEB patients are continuously exposed to the detrimental effects of several pro-inflammatory cytokines and growth factors that alter the transcriptional profile [42], and force fibroblasts to remain into the “myofibroblast state”. Indeed, myofibroblasts chronically reside in the dermis of RDEB patients and contribute to ECM stiffness, an event fueling the fibrotic process in a self-renewing cycle. TGF-β1 signaling plays a key role in establishing and maintaining the fibrotic process in RDEB patients [43], and animal disease models [44,45]. TGF-β1 enhances fibroblast-to-myofibroblast conversion and promotes dermal contractility and ECM stiffness via the activation of both SMAD-dependent and SMAD-independent signaling cascades. The molecular mechanisms underlying the continuous activation of TGF-β signaling in RDEB can be found in the complex series of events mainly driven by COL7 loss, and determining the enzymatic or mechanical release of latent TGF-β1 from ECM-bound complexes. Notably, the matricellular proteins decorin (DCN) [43,46] and thrombospondin 1 (TSP1) are arising as important regulators of TGF-β1 activity in RDEB patients and could represent relevant targets for innovative therapeutic approaches to counteract fibrosis progression [47]. DCN is an interstitial proteoglycan characterized by multiple binding partners and multifaceted activities in the context of ECM [48]. In particular, it plays a key anti-fibrotic role through the blockade of TGF-β1 bioavailability and activation by direct sequestration and indirect mechanisms. DCN expression levels are reduced in RDEB patients and COL7 hypomorphic mice (RDEB mice), negatively correlate with disease severity and strongly affect disease manifestations in RDEB mice (e.g., survival and development of mitten deformities) [43,46]. In addition, numerous studies demonstrate that DCN regulates a variety of cancer-related processes in a context-dependent fashion, playing a dual role of pro- and anti-tumorigenic factor. In the tumor microenvironment, DCN is a potent anti-angiogenetic molecule, and its levels are reduced in the stromal of many solid malignancies [48], including RDEB-associated SCC [49]. Contrarily to DCN, the glycoprotein TSP1 is a TGF-β1 activator up-regulated in fibroblasts from non-tumoral and tumoral stroma of RDEB patients in response to COL7 deficiency [47].

**Figure 2:**
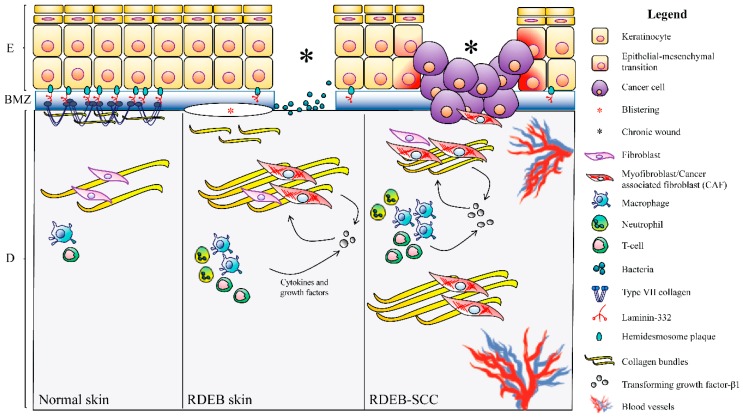
Schematic representation of squamous cell carcinoma (SCC) microenvironment in recessive dystrophic epidermolysis bullosa (RDEB) patients. Left panel. Normal skin. Basal keratinocytes firmly adhere to the basement membrane zone (BMZ) through hemidesmosome protein components (blue ovals) and are also the main producers of laminin-332, an essential component of epithelial BMZs, and of type VII collagen (COL7) that assembles into anchoring fibrils (AFs). AFs extend from the lower part of the BMZ into the upper dermis (papillary dermis), ensuring dermal-epidermal cohesion. Middle panel. In RDEB patients, COL7 deficiency impairs anchoring fibrils formation and leads to skin fragility and blistering (red asterisk) after minor traumas. At sites of chronic blistering, the dermis is enriched in inflammatory cells (neutrophils, macrophages and T-cells) and myofibroblasts: Both cell types produce high amounts of transforming growth factor (TGF)-β1, a master regulator of fibrosis, in an unremitting and self-renewing cycle. In addition, myofibroblasts abundantly produce extracellular matrix components, contributing to dermal stiffening. Chronic wounds (black asterisks) also show high levels of bacterial colonization that contribute to exacerbating inflammation. In the dermis of RDEB patients, the derailed production of cytokines, growth factors and ECM members create the permissive environment for keratinocyte transformation. Right panel. RDEB-SCC microenvironment. Stromal inflammation and fibrosis represent the scaffold for tumor development and progression. Cells with features of cancer-associated fibroblasts (CAFs-like cells) populate tumor stroma and contribute to tumor growth. Keratinocytes undergo epithelial-mesenchymal transition (EMT) and convert to carcinoma cells. SCC microenvironment is characterized by huge inflammation and fibrosis.

A growing body of evidence [50] supports the concept that fibrosis plays a crucial role in SCC development in RDEB patients by creating a permissive tumor microenvironment. Indeed, injury-driven fibrosis and inflammation lead to RDEB fibroblast conversion into cells resembling carcinoma-associated fibroblasts (CAFs) (Figure 2). CAFs represent a heterogeneous cell-type similar to myofibroblast, but able to promote the development of cancer through the production of a set of cytokines, chemokines, signaling molecules and ECM proteins sustaining tumor cells growth and migration [14,51]. Besides their role in wound healing, typical markers of activated fibroblasts/myofibroblasts, such as α-SMA, ED-A fibronectin and TNC, possess a pro-tumorigenic function and their expression levels can be used as prognostic factors in several cancer types [14].

A seminal study published by Ng and coll. in 2012 analyzed gene expression in dermal fibroblasts from healthy subjects, RDEB patients and CAFs from RDEB-SCC and UV-SCC tumor matrices. The mRNA profiling showed that in all disease conditions, genes involved in ECM and cell-adhesion are the most deregulated. In addition, fibroblasts from non-tumor RDEB are characterized by a transcriptome profile similar to that of CAFs from UV-SCC rather that normal fibroblasts, suggestive of a stromal-driven predisposition to SCC development in RDEB patients. On the other hand, gene expression analysis failed to identify a signature of deregulated genes in CAFs from RDEB patients with SCC, but revealed a stepwise progression in gene dysregulation magnitude from healthy fibroblasts to RDEB-SCC CAFs [49]. Proteomic studies confirmed that loss of COL7 in RDEB fibroblasts alters the extracellular proteome and its post-translational modification status [52].

In addition, RNA-seq analysis showed that primary skin fibroblasts from patients affected with three cancer-prone genodermatoses (i.e., KS, RDEB and xeroderma pigmentosum complementation group C, XPC) share a similar transcriptional profile despite the unrelated primary genetic defects [53]. Deregulated genes allow the acquisition of an activated and synthetic fibroblast phenotype resulting in fibrosis and related complications, as tumor growth [53].

The key role of dermal changes in RDEB-SCC pathogenesis has also been highlighted by a proteomic study which evaluated the biological processes commonly deregulated in two high-risk tumors, i.e., RDEB-SCC and metastasizing UV-SCC as compared to low-risk, non-recurrent and non-metastatic, UV-SCC. Quantitative mass spectrometry analysis showed that in RDEB-SCC and metastasizing UV-SCC the proteomic profile is enriched in proteins related to bacterial challenge and ECM remodeling, in accordance with their invasive and metastatic abilities [54]. Specifically, in RDEB-SCC the Gene Ontology (GO) analysis of the differentially expressed proteins showed: (i) The enrichment of terms correlated to tissue inflammation and humoral immunity, two known drivers of tumor transformation [55,56], and (ii) the increased expression of stromal proteins, such as collagen I, XII, XIV and lumican. Notably, a work by Thriene and collaborators investigated the transcriptomic and proteomic profiles of primary keratinocytes from RDEB patients, filling the gap of literature in which studies on the fibroblast contribution in RDEB-related fibrotic processes substantially underestimated the “epithelial” role [57]. Moreover, in this case, alterations in the expression levels of specific ECM components (e.g., genes encoding laminin-332 and LTBP1, respectively down- and up-regulated) appear to be relevant to disease progression. Despite the heterogeneity, due to the inter-individual variability, ECM produced by RDEB keratinocytes includes a cluster of up-regulated proteins related to inflammation and response to wounding, and a cluster of down-regulated proteins made of COL7 interactors [57].

#### 4.3.3. Intracellular Signaling

**Figure 3:**
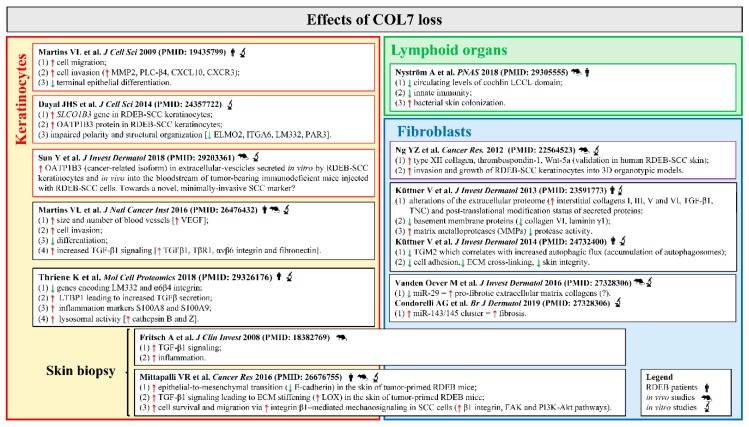
Collagen VII (COL7) deficiency in recessive dystrophic epidermolysis bullosa (RDEB) skin and extracutaneous tissues favors squamous cell carcinoma (SCC) development. The figure summarizes literature findings on relevant pro-tumorigenic processes triggered by COL7 loss in RDEB keratinocytes, fibroblasts and lymphoid organs [42,44,49,52,57,58,59,61,62,63,64,67,84]. Red up arrows indicate increase/up-regulation, green down arrows indicate decrease/down-regulation. Abbreviations: ECM, extracellular matrix; ELMO2, engulfment and cell motility 2; FAK, focal adhesion kinase; ITGA6, integrin subunit alpha 6; LM332, laminin-332; LOX, lysyl oxidase; LTBP1, latent-transforming growth factor beta-binding protein 1; MMP2, metalloproteinase 2; OATP1B3, organic anion transporting polypeptide 1B3; PAR3, partitioning defective 3; PI3K, phosphoinositide 3-kinase; PLC-β4, phospholipase C-β4; RDEB, recessive dystrophic epidermolysis bullosa cutaneous; SCC, squamous cell carcinoma; SLCO1B3, solute carrier organic anion transporter family member 1B3; RDEB-SCC, RDEB-related SCC; TNC, tenascin-C; TβR1, transforming growth factor β receptor 1; TGF-β1, transforming growth factor-β1; TGM2, transglutaminase 2, VEGF, vascular endothelial growth factor.

Notwithstanding the ever-growing number of studies, very little is known on the molecular mechanisms responsible for the development of SCC in RDEB patients and for its unusually aggressive course. As summarized above, the genetic landscape of the RDEB-SCC is similar to that found in the less-aggressive UV-SCC in the general population, and, mutations in the *COL7A1* gene represent the only, major difference between these two tumor types. For this reason, the absence of COL7 in RDEB patients is considered one of the main molecular determinants to disentangle RDEB-SCC behaviors. Several in vitro and in vivo studies contributed to unveil how COL7 loss in RDEB keratinocytes, fibroblasts and extracutaneous tissues leads to a complex series of molecular events determining the progressive cancerization of skin microenvironment via the activation of typical pro-tumorigenic processes, such as inflammation, angiogenesis and tumor cell invasion (Figure 3).

In detail, COL7 loss in non-RDEB SCC keratinocytes enhances migration and invasion, impairs epithelial differentiation, and promotes epithelial-mesenchymal transition (EMT) and vascularization through different mechanisms [58,59], including the activation of TGF-β1 signaling, a known regulator of tumorigenic processes [60]. Indeed, lack of COL7 in non-RDEB-SCC cells (SCC-IC1 cell line) xenografted onto SCID mice determines an increased expression of the active form of TGF-β1, its receptor TβRI, and its downstream targets. Mittapalli and collaborators [61] highlighted the role of COL7 deficiency in carcinogenesis by demonstrating that RDEB mice treated with DMBA/TPA develop skin lesions highly reminiscent of invasive RDEB-SCCs, while wild-type littermates show non-invasive, benign papillomas. The skin of tumor-primed RDEB mice is typified by stiffness and activation of several pro-tumorigenic pathways [61]. COL7 deficiency in RDEB-SCCs impairs front-to-rear keratinocyte polarity in 2D cultures and 3D spheroids through the deregulation of *SLCO1B3*, a gene encoding for the membrane-bound organic anion transport polypeptide OATP1B3, and other members of adhesion complexes [58,62]. In addition, OATP1B3 levels in RDEB-SCCs negatively correlate with COL7 abundance. Of the two OATP1B3 transcripts, the cancer-related isoform (cancer-type, Ct) is up-regulated in variety of cancers, where it modulates the clinical phenotype. Ct-OATP1B3 is overexpressed in RDEB-SCC keratinocytes as compared to non-tumoral RDEB and healthy cells. Recently Ct-OATPB1B3 mRNA has been found in extracellular-vesicles (EVs) released in the culture medium by RDEB-SCC cells and in the bloodstream of tumor-bearing immunodeficient mice upon injection with human RDEB-SCC cells. These findings draw attention to the role of SCC-derived EVs and their molecular cargo in RDEB-SCC pathogenesis, and to their potential usage as diagnostic and prognostic factors of the disease [63].

Recently, it has been reported that COL7 deficiency perturbs keratinocyte lysosomal activity and determines the accumulation of S100A8 and S100A9, two markers of acute and chronic inflammation, into the pathological ECM of RDEB patients. Lack of COL7 also increases the levels of cathepsin B and Z, two lysosomal proteases, and boosts lysosome activity and autophagic flux, all events potentially able to weaken ECM at the BMZ [57]. In contrast, Kuttner and coll. showed that the reduction of transglutaminase 2 (TGM2) lessens autophagic flux in primary fibroblasts from RDEB patients, and correlated this event to the enhanced fibrogenesis [64]. Both in physiological and pathological states autophagy plays a multitude of functions, even opposite, in relation to the cell context. Defective autophagy predisposes cells to different diseases, such as malignant transformation [65], and fibrosis of the skin and other organs [66]. Taken together, these findings indicate a role of lysosomes and autophagy in RDEB pathogenesis and foster further investigations in the field of RDEB-associated SCC.

Finally, microRNAs (miRNAs), a class of small non-coding RNAs with pleiotropic functions, are emerging as novel players in RDEB fibrosis [42,67], and potential regulators in tumor stroma cancerization. Deregulation of miRNAs expression and activity has been demonstrated to play a significant role in a variety of human diseases, including fibrotic skin disorders and SCC [68,69], but their involvement in RDEB and its complications are almost unexplored. Recently, we demonstrated the pro-fibrotic role of miR-145-5p in primary skin fibroblasts from RDEB patients (RDEBFs). In RDEBFs, miR-145-5p is up-regulated as compared to cells from healthy subjects, and its inhibition determinates the reduction of typical fibrotic behaviors (i.e., contractile force, proliferation and migration) and fibrotic markers by direct and indirect modulation of multiple and partially overlapping signaling cascades, such as the NOTCH pathway [42].

#### 4.3.4. Inflammation

An increasing amount of experimental evidence, obtained in animal disease models and RDEB patients, pinpoints the relevance of inflammatory processes in EB pathomechanisms and disease complications. An imbalance in cytokine levels has been described in vitro in cells derived from EB animal models and patients, as well as in vivo and suggests that inflammation deeply alters the dermal microenvironment, and at the same time, contributes to worsening systemic disease manifestations. In detail, COL7 hypomorphic mice show an unremitting inflammatory state in the upper dermis [44], and *col7*−/− mice display increased serum concentrations of the pro-inflammatory cytokine interleukin (IL)-6 [70]. Moving towards RDEB patients, a study of Esposito and coll. demonstrated that circulating levels of antibodies against skin proteins and pro-inflammatory cytokines, in particular, IL-6, are significantly higher with respect to healthy controls, and correlate with disease severity [71]. Of note, IL-6 levels were significantly associated with EB extension (localized or generalized disease), disease severity and anti-skin antibodies levels [71]. Accordingly, comparative analysis of primary fibroblasts from a couple of RDEB monozygotic twins with different disease manifestations showed increased levels of IL-6 in conditioned medium of fibroblasts derived from the individual with the more severe phenotype [43]. IL-6 has been implicated in the pathogenesis of systemic scleroderma (SSc), an autoimmune disease leading to fibrosis of the skin and internal organs, and of fibrosis in two mouse models, the bleomycin model (BLM) and the tight-skin mouse (Tsk-1). IL-6 signals through STAT3, a transcription factor up-regulated in SSc patients and BLM/Tsk-1 mice and able to control fibroblast-to-myofibroblast conversion, in cooperation with TGF-β [72].

Beyond its well-known role in fibrosis, IL-6 mediates cross-talk between CAFs and tumor cells [73], and represents a key player in the growth and metastatic evolution of several epithelial tumors, such as head and neck SCC and esophageal SCC [74,75,76]. Interestingly, preliminary findings show that the protein signal transducer and activator of transcription 3 (STAT3), a downstream effector of IL-6, is constitutively activated in untransformed RDEB-derived keratinocytes and in RDEB-derived SCC, both in basal conditions and after stimulation with TGF-β [70]. The hyper-activation of the IL-6 signaling cascade could at least partly explain the increased risk of RDEB patients to develop aggressive SCCs, and, in turn, represents a novel prognostic and therapeutic target in RDEB-SCC [72,77].

High mobility group box 1 (HMGB1) is a non-histone DNA binding protein with multifaceted functions in relation to the context. Secreted HMGB1 exerts a cytokine-like function, regulating inflammatory state and immunity by different modalities. HMGB1 also has a tumor-promoting role and has been found at high levels in different types of cancer, including SCC [78,79,80]. Serum levels of HMGB1 are elevated in RDEB patients and correlate with disease severity [81]. In RDEB, circulating HGMB1 is likely released by COL7-deficient keratinocytes in response to skin injury with the aim to recruit bone marrow cells at skin lesional sites and promote epithelial regeneration [82]. In accordance with the elevated serum levels and pro-inflammatory function of its extranuclear form, cytosolic HMGB1 is strongly up-regulated in RDEB lesional skin and even more in RDEB-SCC as compared to control skin [83]. Finally, the observation that TLR5, the leukocyte receptor for flaggelin, induces HGBM1 in a mouse model of wound-induced cancer introduces a recently identified topic in RDEB-SCC development: The microbial infection of the wound [83].

#### 4.3.5. Microbial Infection

Experimental evidence suggests a relation between inflammatory processes, microbial infection and SCC development [83]. A typical feature of skin and mucosal lesions in RDEB patients is their colonization with *Staphylococcus aureus* and one or more additional commensal bacteria [84,85]. The presence of large chronic wounds in RDEB patients favors bacterial colonization, but it cannot represent the only cause of the high bacterial burden in RDEB patients; additional factors, such as an impaired immunity response in RDEB patients, must be involved in the aberrant wound microbial colonization. Indeed, non-RDEB patients with large, severe burn wounds resembling those of RDEB patients display a considerably lesser infection rate by *Staphylococcus aureus* as compared to RDEB patients [85,86]. In addition, RDEB mice show an elevated *Staphylococcus aureus* colonization of the unwounded skin and an increased, though ineffective, antimicrobial response as compared to wild-type mice [84]. Nyström and coll. recently demonstrated that the increased susceptibility to bacterial colonization in RDEB wounds is independent of skin integrity, but results from the absence of COL7 in the ECM of lymphoid conduits of spleen and lymph nodes. In lymphoid conduits, COL7 binds and sequesters cochlin (COCH), a modulator of innate immunity. In response to bacterial infection, COCH is processed by aggrecanase to release the circulating LCCL domain, which activates macrophages and neutrophils and stimulates bacterial clearance at infection sites. Thus, COL7 loss in lymphoid ECM of RDEB patients impairs COCH localization and determines a reduction of the LCCL domain, an event that results in an increased bacterial burden [84] (Figure 3).

In contrast, human papillomaviruses (HPV) infections, that represent a well-known risk factor for mucosal and cutaneous SCC development in the normal population, do not seem related to SCC onset in RDEB patients [87].

#### 4.3.6. Immunity

The relation between immunity and cancer is well-established. Cancer cells express an abnormal set of proteins or abnormal levels of normal cellular proteins that can function as tumor antigens and can be detected and eliminated by immunosurveillance. At the same time, some cancer cells typified by low immunogenicity can get away from the immunosurveillance, and, during a more or less long period are shaped by their intrinsic genetic instability and by dynamic interactions with immune cells to, then, proliferate and create a permissive tumor microenvironment. Given the role of immunity in selecting and “sculpting” tumor cells, this process is defined as immunoediting. Several studies have described the role of the immune system in cutaneous SCC development [88], but knowledge about the role of immunity in RDEB-SCC is scanty and mainly indirect. Within this fragmented scenario, Riihilä and coll. recently described the up-regulation of the complement system members C1r and C1s in non-RDEB-SCC and RDEB-SCC compared to normal skin, in situ SCCs and actinic keratoses, and demonstrated their role in cell viability, apoptosis resistance and migration [89]. Of note, the augmented C1s staining in RDEB tumor cells could correlate with the activation of phosphoinositide 3-kinase (PI3K) and mitogen-activated protein kinase (MAPK) pathways, and contribute to the elevated migratory and metastatic abilities of cancerous cells in RDEB patients [89].

### 4.4. Therapeutic Strategies: The Present and the Future

#### 4.4.1. Current SCC Therapies

According to current best clinical practice guidelines for EB-SCC treatment, a strict follow-up of skin wounds and scars, biopsies of clinically suspicious lesions and wide local surgical excision of tumors represent the standard of care in EB patients [26]. On the other hand, there is no evidence that radiotherapy and conventional chemotherapy are definitively effective, and their side effects may overweight benefits in these fragile patients. Thus, they are only recommended as palliative modalities for locally advanced inoperable and metastatic EB-SCC [26]. In addition, the transitory progression-free disease has been reported in very few RDEB patients with locally advanced or metastasized SCCs treated with cetuximab, a monoclonal antibody against epidermal growth factor receptor (EGFR) approved in Europe and USA as an adjuvant treatment for locally-advanced and metastasized head and neck SCCs [90,91,92,93]. On the other hand, the complexity of oncologic therapies in RDEB-SCC is well-testified by the ineffectiveness and even the paradox effect on new SCCs development of immunotherapy with an anti-PD1 molecule, pembrolizumab, which instead is FDA-approved for advanced progressing head and neck SCC [93]. Overall, current figures which report a mean survival time after the first SCC of 4 years in RDEB-SG clearly show the urgent need of novel, more effective therapeutic approaches for SCCs in RDEB patients [94].

#### 4.4.2. Therapeutic Perspectives

The clinical and experimental evidence, concerning the pivotal role of injury- and inflammation-driven fibrotic process in severe EB complications, focused scientific and clinical EB community efforts on anti-fibrotic and anti-inflammatory therapeutic strategies able to lessen disease manifestations (symptom-relief therapies). Of course, the most severe, cancer-prone EB subtypes (RDEB and JEB) represent the main targets of experimental drugs aimed at contrasting disease symptoms, and, in turn, at delaying SCC onset.

(1) Symptom-relief therapies

As for RDEB, the current anti-fibrotic treatment approaches mainly converge in the attenuation of TGF-β signaling cascade, and the majority is under investigation at the preclinical level [45,46,95]. On the other hand, losartan, a repurposing drug already approved for the treatment of hypertension, represents the most advanced investigational molecule for the treatment of RDEB fibrosis and entered a phase I/II clinical trial [45] (Reflect study, EudraCT no. 2015-003670-32). Though losartan’s anti-fibrotic properties have been demonstrated in primary fibroblasts from RDEB patients and in RDEB mice [45], it is too early to draw up a balance about its efficacy and safety in fragile RDEB patients. However, regardless of its outcome, the “losartan experience” focuses our attention on the relevance of drug repositioning as a fast, poor-risk, and efficient approach to find and propose novel therapeutic approaches for rare diseases, such as EB, with an urgent need for therapies [96].

(2) Curative therapies

Alongside the above symptom-relief therapies, curative interventions based on molecular (gene- and protein-therapy), and cellular approaches have been developed.

(i) Gene therapies

Gene therapies aim at replacing or correcting disease-causing gene mutations in ex vivo patient cells, including induced pluripotent stem cells (iPSCs) [97], fibroblasts [98,99], and keratinocytes with high growth potential, termed holoclones. Different strategies ranging from retroviral-mediated gene transfer to genome editing (e.g., TALENs and CRISP/CAS9 systems) [100,101,102,103,104,105] can be used for gene correction in patient cells. In the approaches which are already in trials, primary keratinocytes from patients are transduced in vitro with retroviral vectors encoding a normal protein, expanded in the laboratory, and transplanted as gene-corrected epidermal sheets (i.e., autologous cultured epidermal grafts) in patients [102,103,104,105]. These therapeutic interventions entered clinical trials for RDEB (ClinicalTrials.gov Identifier: NCT01263379 and NCT02984085) [103,104], and JEB (ClinicalTrials.gov Identifier: NCT02984085) with exciting results in the latter [105]. However, viral-based strategies for gene correction encompass experimental challenges, e.g., methodology for gene delivery, editing at off-target sites and duration of the therapeutic effects [102,103,104,105], and safety issues such as the risk of malignancies, due to adverse mutagenic events.

(ii) Cell therapies

Cell-based therapies aim at restoring dermal-epidermal adhesion mainly through intradermal/intravenous injections of the following healthy allogeneic cell-types: (i) Fibroblasts, (ii) mesenchymal stromal stem cells (MSCs), and (iii) bone marrow (BM)-derived stem cells. They are able to localize in the skin and correct the disease-specific biochemical defect, by producing COL7. Intradermal injection of wild-type fibroblasts and MSCs in RDEB mice increases COL7 along cutaneous BMZ and improves skin integrity and resistance to mechanical forces with minimal adverse effects [106,107]. Similarly, preclinical studies showed that BM cells infused in RDEB mouse model migrate to sites of injury and contribute to lessening disease manifestations [108,109]. As for other cells with stem functionality, human umbilical cord blood-derived unrestricted somatic stem cells (USSCs) have shown anti-fibrotic effects and amelioration of disease phenotype in *col7a1*−/− mice through the up-regulation of two relevant TGF-β1 antagonists: DCN and TGF-β3 [110,111,112]. However, pilot cell therapy trials on RDEB patients revealed modest to absent clinical efficacy and improvement in patient’s quality of life, low-tolerability or severe side effects [113,114,115,116,117].

(iii) Protein- and RNA-based therapies

Protein-based therapeutics (PBTs) have their roots in the immediate advantage to administer to the patient, regardless of cell- and vector-based delivery, the correct form of the defective protein. The major problem of PBTs is their immunogenicity: The tendency to generate an immune response against the exogenous proteins, with loss of effectiveness and potential systemic complications. As for RDEB, the recombinant type VII procollagen (PCOL7) has been successfully intradermal/intravenous injected in *col7a1*−/− mice [118]. PBTs in RDEB patients are challenging, due to the high amount of clinical-grade recombinant protein needed for lifelong administration and its potential immunogenicity [119]. However, some interesting answers are expected from PTR-01: A human recombinant COL7 for the treatment of RDEB that recently entered a phase I/II clinical trial (ClinicalTrials.gov Identifier: NCT03752905).

RNA-based therapies (RBTs) can be applied for RDEB patients bearing *COL7A1* mutations in specific in-frame exons, whose deletion does not lead to major structural changes at the protein level. In vitro and in vivo preclinical studies demonstrated that mutated exons could be skipped/deleted by antisense oligonucleotides (AONs) leading to the synthesis of a COL7 protein, similar to the wild-type, but lacking the defective region [120,121]. A phase I/II multicenter clinical trial is assessing safety and effects of the topical administration of QR-313, an AON determining the exclusion (skipping) of exon 73 from COL7A1 mRNA, in DEB patients bearing at least one pathogenic mutation in exon 73 (ClinicalTrials.gov Identifier: NCT03605069).

(iv) PTC read-through strategy

Another curative approach for RDEB and JEB patients bearing nonsense mutations consists in forcing premature termination codons (PTCs) read-through by the topic or systemic administration of molecules with non-sense mutations suppression activity, such as aminoglycoside antibiotics (e.g., gentamycin B1) [122] or specific anti-inflammatory drugs (e.g., amlexanox) [123]. Of note, gentamycin B1 treatment of RDEB patients has been investigated at a clinical level, with encouraging results (ClinicalTrials.gov Identifier: NCT03012191). Antibiotic toxicity and immunogenicity of the newly-formed COL7 are the main potential drawbacks of this type of intervention.

(3) SCC-targeted therapies

The omics-sciences and the strong translation approach in the EB research field represent the ground of an experimental ferment aiming to identify novel deregulated pathways and therapeutic targets in RDEB-SCC. Among them, polo-like kinase-1 (PLK-1) is emerging as a promising candidate. PLK-1 is a member of the serine/threonine protein kinases family, which has important roles in the mitotic process; and its over-expression is a common feature in a great number of tumors types, including RDEB-derived SCC [124]. For these reasons, PLK-1 represents a well-established target for cancer therapy [125]. Recently, Atanasova and coll. demonstrated that rigosertib, a PLK-1 inhibitor, exerts a strong and selective pro-apoptotic role on RDEB-derived SCC keratinocytes [126]. These experimental findings supported rigosertib admission to a phase II clinical trial to evaluate its safety and efficacy in RDEB patients with unresectable/standard care unresponsive, locally advanced or metastatic SCC (ClinicalTrials.gov Identifier: NCT03786237).

## 5. Junctional EB

### 5.1. Clinical Features

Junctional epidermolysis bullosa (JEB) is less common than DEB and EBS: Its incidence has been estimated just over 2 per million live births in the USA [127]. The two commonest JEB subtypes, JEB generalized intermediate (JEB-GI) and JEB generalized severe (JEB-GS), are recessively-inherited and due to mutations in any of the three genes, *LAMA3A*, *LAMB3*, *LAMC2*, encoding the three chains of the major epithelial laminin isoform, laminin-332 (LM332), or, for JEB-GI, to mutations in the *COL17A1* gene encoding a structural component of hemidesmosomes, collagen XVII (COL17), also known as 180-kD bullous pemphigoid antigen (BP180). JEB-GS is early lethal, usually within the first 12 months, due to extensive skin and mucosal involvement leading to failure to thrive, upper airway obstruction and sepsis [128]. It is characterized by mutations resulting in a complete lack of LM332, which is essential for adhesion of stratified and also simple epithelia. On the other end, JEB-GI is compatible with life and associated with variably reduced LM332 amounts or with absent or reduced COL17, which is expressed in stratified epithelia. JEB-GI presents with phenotypes of variable severity as to the extent of skin and mucous membrane involvement. In adulthood, development of chronic wounds which heal with atrophic scarring is typical. Data from small patient cohorts have suggested that adult JEB patients with defective LM332 are at increased risk of developing SCC starting from their third decade of life [129].

### 5.2. LM332 and COL17 in SCC in the General Population

LM332 is a multidomain glycoprotein and the major adhesion ligand of epithelial cells. In the skin, it is synthesized and assembled as high-molecular-weight heterotrimeric precursor within the endoplasmic reticulum of basal keratinocytes. The LM332 heterotrimer is composed of α3A, β3 and γ2 polypeptides, encoded by *LAMA3A*, *LAMB3*, *LAMC2* genes, respectively. The precursor molecule is secreted and deposited into the ECM, where the α3 and γ2 chains undergo proteolytic maturation to smaller forms. C-terminal processing of the α3 chain can be mediated by different enzymes and consists of cleavage of the laminin globular (LG) domains 4 and 5 (LG45) within the linker region between LG3 and LG4 [130]. Outside the cell, LM332 simultaneously binds cell surface receptors and ECM components, such as integrins α6β4 and α3β1, syndecans-1 and -4, COL17 and COL7, exerting a critical role in skin integrity, as well as in multiple biological processes, including keratinocyte survival and migration [24]. Important functions have been assigned to the α3 chain and its processing, in both physiological and pathological conditions.

The processed LM332 lacking LG45 (LM332-α3_165_) is mainly found in mature BMZs, where it orchestrates the formation of anchoring structures through α3β1 and α6β4 interactions [130]. Increased synthesis and processing can be detected in chronic wounds in response to inflammation and infection [131]. In contrast, LM332 with unprocessed LG45 (LM332-α3_200_) is detectable in migratory/remodeling situations, such as wound repair [132], and in SCCs from the general population [133].

Importantly, lack of LM332 halts SCC tumorigenesis of HRAS/IkBα-transformed human epidermis grafted onto immunodeficient mice, while restoration of its expression in the same model raises SCC tumorigenesis [134]. In this process, LM332 interactions with its ECM ligand COL7 and cell receptor integrin α6β4 are crucial for tumor invasion via activation of PI3K/AKT pro-tumorigenic signaling [135,136]. Subsequently, in vitro and in vivo studies revealed that the LG45 subdomain of LM332-α3_200_ promotes invasion of transformed human keratinocytes by activating the matrix metalloproteases MMP-9 and MMP-1, and triggers PI3K and ERK pathways [133,137]. Interestingly, targeting LG45 with a specific antibody counteracts tumorigenesis in vivo [133].

The role of LM332 in cancer is also illustrated by its ability to promote CAFs differentiation and maintenance [138], as well as tumor spreading as shown by the presence of specific LM332 chains, mainly the γ2, at the leading edge of invading carcinomas and their relationship with tumor invasiveness and patient prognosis [139,140,141]. However, it remains to be clarified if the increased staining of specific LM332 chains in cancer specimens reflects a disease-specific mechanism of synthesis and processing.

Interestingly, COL17 is also enhanced in carcinogenesis similarly to its ligand LM332 [142]. Increased expression and shedding of its ectodomain from the cell surface have been observed at the tumor-stroma interface during SCC invasion and metastasis, while shedding inhibition prevents SCC progression [142].

### 5.3. LM332 and COL17 in SCC in JEB Patients

Since the expression of LM332 and COL17 positively correlates to tumorigenesis of non-EB SCC the role of LM332 and COL17 in JEB-related SCC tumorigenesis is not easily interpretable: In JEB-GI patients with *COL17A1* mutations COL17 is often absent, and in JEB-GI patients with mutations in either *LAMB3*, *LAMC2* and *LAMA3* genes LM332 expression is reduced. Nevertheless, data from case reports and case series indicate that adult JEB patients have an increased risk (1:4) of developing SCC starting from their third decade of life [18,129,143]. Reported cases more frequently harbor mutations in genes encoding LM332 chain subunits, more rarely in *COL17A1*. The first SCC develops at a younger age compared to non-EB individuals [18]. They can be multiple, histologically well or moderately differentiated, and can have an aggressive course. Notably, SCCs almost exclusively arise on lower extremities mostly in the pretibial region and within areas of chronic blistering, long-standing erosions/ulcers, or atrophic scarring [129]. This suggests that in JEB, as in RDEB, chronic wounds induced by repeated mechanical traumas lead to tissue inflammation, subsequent ECM remodeling/dermal fibrosis and skin microenvironment alterations fueling SCC development and recurrence (see above) [54]. However, research in these fields, at least with regard to JEB, is almost lacking.

The pathogenesis of SCCs might also be related to the induction of cell migration and/or increased integrin-mediated signaling consequent to LM332 reduced levels and altered functions. Indeed, the amount of deposited LM332 inversely correlates with the rate of keratinocyte migration [144]. Notably, a reduction of LM332 is detected in SCC developed in RDEB individuals [57]. Lack of COL17 also enhances both keratinocyte propensity to migrate and PI3K signaling [145,146]. Primary keratinocytes from a JEB-GI patient with a naturally occurring mutation that truncates the LG45 subdomain increase their migration in vitro [143]. In this patient, the secreted and deposited mutant LM332 from skin and keratinocytes is reduced by about 50%. Interestingly, this individual developed an extensive number of keratoacanthomas and well-differentiated locally invasive SCCs, which did not metastasize over 20 years. Thus, the maintenance of sufficient amount of protein (≈ 50% or more) together with its qualitative defects might allow intrinsic pro-tumorigenic properties of LM332 to be conveyed, promoting SCC progression and recurrence. This case study, however, indicates that LM332 with truncated LG45 promotes, rather than inhibit, cell migration. Overall, these data clearly show the need for further investigations of the effects on cell signaling by LM332 mutations associated with SCC tumorigenesis in humans.

## 6. Kindler Syndrome

### 6.1. Clinical Features

Kindler Syndrome (KS) is the rarest EB type, with a few hundred patients described worldwide. It is caused by biallelic mutations in the *FERMT1* gene that encodes for kindlin-1, a cytoplasmic component of focal adhesions involved in integrin signaling and linkage of the actin cytoskeleton to the ECM [147]. The majority of *FERMT1* mutations lead to premature termination of translation and to loss of the kindlin-1 protein [147]. In addition to skin fragility, the hallmark of the disease is photosensitivity not present in other EB types. With advancing age, KS patients show an improvement skin blistering, but develop progressive and generalized skin atrophy and a mixture of skin atrophy, dyspigmentation and telangiectasia, known as poikiloderma, at photoexposed areas (face and neck), as well as hand and foot pseudosyndactyly [147].

Several case reports and a case series indicate that KS patients in adulthood have an increased susceptibility to SCC development [147,148,149]. Recently, Guerrero-Aspizua and coll. analyzed a cohort of 91 KS patients, 69 previously published [147,149], and 22 unpublished cases, in order to evaluate the incidence of SCC in KS syndrome at different ages [150]. 14.3% of the patients (13 out of 91) developed 1 or more well-differentiated SCC, for a total of 26 SCCs (25 in the skin and 1 in the oral mucosa). Cumulative risk of developing at least one SCC for patients with KS increases with age, and reaches the 66.7% by age 60. Seven out of 13 KS patients with SCC presented metastases. Similar to other EB-related SCC, KS-SCCs are aggressive and represent the cause of death in 38.5% of patients [150].

### 6.2. Pathways Involved in KS-Related SCC Development

As for KS, the molecular mechanisms underlying SCC development are very peculiar, since KS represents the only EB type in which a contribution to tumor onset could be given by UV-induced photodamage. Emmert and coll. [151] demonstrated that loss of kindlin-1 in SCC cells from a mouse model determines an unbalanced endogenous oxidative state, as shown by the reduced glutathione/glutathione disulphide ratio (GSH/GSSG ratio) and by the increased levels of reactive oxygen species (ROS) as compared to wild-type, kindlin-1 expressing SCC cells. Absent kindlin-1 sensitizes keratinocytes to oxidative stress- and UV-induced damage, determining an impaired activation of the ERK pathway. In addition, preliminary findings show that in primary human keratinocytes, kindlin-1 deficiency leads to cyclin-dependent kinase-1 (CDK-1) inhibition, and DNA damage in response to oxidative stress [152]. In the context of cancer, ROS have been reported to have both pro- and anti-survival functions, but the possible relation between SCC onset in KS patients and ROS-induced mutagenesis following UV exposure remains to be established.

Keratinocytes from KS patients exhibit premature senescent features [153]. Senescent cells may modify stromal microenvironment and influence the redox state of neighboring cells through paracrine signaling [154]. Notably, senescence-associated with oxidative damage could represent a tumor-promoting mechanism in epithelial cells [155]. Recently, Michael and coll. demonstrated that the absence of kindlin-1 in primary keratinocytes from KS patients is responsible for the increased targeting of EGFR for lysosomal degradation. This process leads to a marked reduction in EGFR protein levels, its mislocalization, and an impaired response to EGF stimulation, as shown by the decreased phosphorylation of EGFR and its downstream target ERK1/2 [156]. In keratinocytes, the attenuation of EGFR signaling cascade has implications in multiple biological processes, such as migration [156], immunity [157], and inflammation [158].

On the other hand, the pathomechanisms responsible for SCC development in KS patients could be recapitulated at least in part by fibrosis- and inflammation-driven alterations in the stromal microenvironment similar to those described in the other EB-derived SCC. Indeed, in vitro studies revealed that KS keratinocytes express increased amounts of growth factors and pro-inflammatory cytokines, in particular, IL-20 and IL-24, in response to stress agents, such as UVB irradiation [159]. Soluble factors secreted by KS keratinocytes target dermal fibroblasts and activate them to express α-SMA and to produce high amounts of collagen I and tenascin [159]. Of note, this fibrotic and inflammatory background was confirmed in KS skin in vivo [159]. In addition, loss of kindlin-1 in a mouse model of KS promotes αvβ6 integrin–mediated TGF-β activation and inhibits Wnt–β-catenin signaling, enlarging different stem cell (SC) compartments and increasing SC proliferation [160].

Finally, preliminary findings on molecular features and genetic profiles of 48 SCCs from patients affected with RDEB (*n* = 10), JEB (*n* = 1) and KS (*n* = 7) [161] show a common molecular signature in all SCCs samples. EB-related SCC were typified by the up-regulation of EGFR and cytochrome c oxidase subunit II (COX2), a marker of inflammation, and by the expression of at least one immune checkpoint among CTLA-4, PD-1 and PD-L1. Mutational signatures resulted very similar between EB-SCCs and UV-SCCs. However, KS-SCCs showed mutational burden and profiles distinct from those found in RDEB-SCCs [161]. Overall these findings point to the existence of partly shared pathomechanisms in EB-SCC development which could be relevant for the identification of common therapeutic targets.

## 7. Conclusions

Inherited EB is a group of rare and life-threatening skin blistering disorders, for which no curative therapies are still available. The most severe EB subtypes expose patients to highly disabling disease complications, including the development of aggressive cutaneous SCCs at lesional skin sites (EB-SCCs). In RDEB patients, SCCs are recurrent, metastasizing and therapy-resistant and represent the first cause of death and reduced life expectancy in these fragile subjects. The unique behaviors and the adverse outcome make RDEB-SCC the most investigated EB-related tumor at the expense of JEB- and KS-SCC, which are poorly explored both clinically and molecularly. As for RDEB, the last ten years of basic research and omics-studies (e.g., genomics, transcriptomics and proteomics) in primary cells from patients, skin biopsies and mice models revealed the key role of chronic tissue damage in creating a permissive tumor microenvironment and brought out a consistent number of molecules deregulated in RDEB-associated fibrosis and inflammation. However, despite the growing amount of data, the knowledge scenario on RDEB-SCC is often not completely informative as the validation of results in tumor models is missing. Alongside the need for better understanding genetics and molecular bases of all EB-SCCs, also in view to obtain efficient and patient-tailored therapies, resources and efforts should be directed on the already got findings, planning long-lasting, multidisciplinary and translational SCC-focused studies.

In conclusion, we highlight the potentially relevant impact of the findings concerning (**i**) the mutagenic process driven by APOBEC family members in response to chronic tissue damage; (**ii**) the role of *NOTCH1* mutations/NOTCH pathway in SCC development; (**iii**) the action of inflammatory mediators, in particular, IL-6, in tumor progression and spreading; (**iv**) the impact of wound bacterial colonization and immunity in carcinogenesis; (**v**) the use of circulating molecules and extracellular-vesicles as novel, minimally-invasive diagnostic and prognostic factors of the disease.

## Figures and Tables

**Table 1:** Literature findings on cross-comparison of significantly mutated genes in recessive dystrophic epidermolysis bullosa (RDEB)-squamous cell carcinoma (SCC) and cutaneous SCC in the general population.

		Significantly Mutated Genes																			
Reference	Disease Model (Sample Size)	*CASP8*	*NOTCH1*	*TP53*	*CDKN2A*	*FAT1*	*ARID2*	*HRAS*	*KMT2B*	*ARHGEF6*	*FAM114A2*	*LRRC8A*	*PHF13*	*SPTBN4*	*NOTCH2*	*SMARCA4*	*EGFR*	*NF2*	*NOTCH4*	*PRDM9*	Other Genes
Cho R.J. et al. *Sci. Transl. Med. 2018* (PMID: 30135250) [31]	RDEB SCCs (*n* = 31)	**38.7**	**54.8**	**45**	**32.2**	**22.5**	**12.9**	**12.9**	**19.3**												
Sans-DeSanNicolas L. et al. *J. Invest. Dermatol. 2018* (PMID: 29291383) [30]	RDEB SCC1 (*n* = 1)									**+**									**VAF 20%**	**VAF 15.9%**	
	RDEB SCC2 (*n* = 1)		**+**								**+**	**+**	**+**	**+**	**VAF 36.47%**					**VAF 9.02%**	
Inman G.J. et al. *Nat. Commun. 2018* (PMID: 30202019) [7]	non-EB SCCs (*n* = 40)		**75**	**70**	**45**			**22.5**							**50**						*ATP1A1, CACNA1C, CLCN3, CRY1, FLNB, GLIS3, GRHL2, HERC6, LCLAT1, MAP3K9, MAPK1IP1L, PTEN, SF3B1, TMEM51, TRAPPC9, VSP41, WHSC1.*
Li Y.Y. et al. *Clin. Cancer. Res. 2015* (PMID: 25589618) [33]	metastatic SCCs (*n* = 29)	**N/P**	**48**	**79**	**45**	**N/P**			**N/P**	**N/P**	**N/P**	**N/P**	**N/P**	**N/P**		**28**	**14**	**17**		**N/P**	
Pickering C.R. et al. *Clin. Cancer. Res. 2014* (PMID: 25303977) [32]	aggressive UV-induced SCCs (*n* = 39)	**23.1**	**59**	**94.9**	**43.6**	**43.6**		**20.5**							**51.3**						*AJUBA, BBS9, BF2D, COBLL1, DCLK1, DCLRE1A, FBX021, KMT2C, OPN3, PARD3, PEG10, RASA1, RBM46, SEC31A, SNX25, ZNF644.*

[31]: Significantly mutated genes with a false discovery rate (FDR) *q* < 0.001 after analysis with MuSiC algorithm are indicated. Mutated genes are shown from the most (*CASP8*) to the least (*KMT2B*) significant. A different statistical analysis performed using MutSigCV algorithm identified only three genes (*CDKN2A*, *CASP8*, and *TP53*) as significantly mutated. [30]: The sign plus (+) indicates the genes mutated in two RDEB-SCCs, RDEB-SCC1 and RDEB-SCC2, with variant allele frequency (VAF) > 45%. The complete list of significantly mutated genes with VAF > 20% is available in Ref. 30 as supplementary material. Values within the cells indicate VAF for NOTCH pathway members and *PRDM9*; the only mutated gene shared between RDEB-SCC1 and RDEB-SCC2. [7]: Significantly mutated genes were identified by at least two out of three algorithms used (MutSigCV, Oncodrive-FM and Oncodrive-CLUST). [33]: Targeted sequencing using the OncoPanelv2 platform. Significantly mutated genes were determined by MutSigCV (*q*-value ≤ 0.1). *N*/*P* = Not Profiled. [32]: Genes were identified as significant by MutSigCV algorithm or by at least two out of three other algorithms.

## Abbreviations

EB	Epidermolysis bullosa
BMZ	Basement membrane zone
RDEB	Recessive dystrophic epidermolysis bullosa
JEB	Junctional epidermolysis bullosa
KS	Kindler syndrome
SCC	Squamous cell carcinoma
COL7	Type VII collagen
ECM	Extracellular matrix
TNC	Tenascin-C
TGF-β1	Transforming growth factor-β1
DCN	Decorin
EMT	Epithelial-mesenchymal transition
IL-6	Interleukin-6
LM332	Laminin-332
COL17	Type XVII collagen
LG45	Laminin globular domains 4 and 5
JEB-GI	Junctional epidermolysis bullosa, generalized intermediate subtype
